# The Regulatory Networks That Control *Clostridium difficile* Toxin Synthesis

**DOI:** 10.3390/toxins8050153

**Published:** 2016-05-14

**Authors:** Isabelle Martin-Verstraete, Johann Peltier, Bruno Dupuy

**Affiliations:** 1 Laboratoire Pathogenèse des Bactéries Anaérobes, Department of Microbiology, Institut Pasteur, 25 rue du Dr Roux Paris, Paris 75015, France; isabelle.martin-verstreate@pasteur.fr (I.M.-V.); johann.peltier@pasteur.fr (J.P.); 2 UFR Sciences du vivant, University Paris Diderot, Sorbonne Paris Cité, Cellule Pasteur, Paris 75015, France

**Keywords:** *C. difficile*, PaLoc, toxin regulation, stress signals, carbon catabolite repression system, growth phase, sporulation, c-di-GMP, quorum sensing

## Abstract

The pathogenic clostridia cause many human and animal diseases, which typically arise as a consequence of the production of potent exotoxins. Among the enterotoxic clostridia, *Clostridium difficile* is the main causative agent of nosocomial intestinal infections in adults with a compromised gut microbiota caused by antibiotic treatment. The symptoms of *C. difficile* infection are essentially caused by the production of two exotoxins: TcdA and TcdB. Moreover, for severe forms of disease, the spectrum of diseases caused by *C. difficile* has also been correlated to the levels of toxins that are produced during host infection. This observation strengthened the idea that the regulation of toxin synthesis is an important part of *C. difficile* pathogenesis. This review summarizes our current knowledge about the regulators and sigma factors that have been reported to control toxin gene expression in response to several environmental signals and stresses, including the availability of certain carbon sources and amino acids, or to signaling molecules, such as the autoinducing peptides of quorum sensing systems. The overlapping regulation of key metabolic pathways and toxin synthesis strongly suggests that toxin production is a complex response that is triggered by bacteria in response to particular states of nutrient availability during infection.

## 1. Introduction

The genus *Clostridium* comprises a vast and heterogeneous group of rod-shaped, spore-forming, Gram-positive and obligate anaerobic bacteria [1]. Members of this genus are widespread in the environment, and the majority of species are considered to be saprophytic, while some species are pathogenic and responsible for a wide spectrum of diseases in both humans and animals. The pathogenic clostridia cause a variety of neurotoxic, histotoxic and enterotoxic diseases, which are associated with the production of potent exotoxins in most cases [2]. Among the enteric clostridia, *Clostridium difficile* (recently renamed *Peptoclostridium difficile* [3]) is the major cause of nosocomial diarrhea in adults with a compromised gut microbiota caused by the use of broad-spectrum antibiotics [4]. The symptoms of *C. difficile-*associated disease range from mild diarrhea to severe life-threatening pseudo-membranous colitis and toxic megacolon associated with high mortality rates [5]. These symptoms are essentially caused by the production of two exotoxins: TcdA and TcdB. These toxins glycosylate Rho family GTPases in host cells, leading to the disruption of the actin cytoskeleton, cell death and a strong inflammatory response [6]. Toxins A and B are both members of the large clostridial toxins (LCT) family, which includes the lethal and hemorrhagic toxins (TcsL and TcsH, respectively) of *Clostridium sordellii*, the α toxin (TcnA) of *Clostridium novyi* and TpeL from *Clostridium perfringens* isolates from domestic livestock [7]. In addition, approximately 20% of *C. difficile* isolates obtained in non-outbreak situations [8] also produce a third toxin that is named binary toxin (CDT) [9]. This toxin consists of two polypeptides: a binding component, CDTb, responsible for attachment of the toxin complex to the host cell surface [10] and the active component, CDTa that displays an actin-specific ADP-ribosyltransferase activity. The *C. difficile* CDT is closely related to the iota toxin from *C. perfringens* type E, CST binary toxin from *Clostridium spiroforme* and C2 toxin from *Clostridium botulinum* [11] The role of CDT in the disease process is still a matter of debate since many virulent strains do not produce CDT. However, it has been shown that CDT depolymerizes the actin cytoskeleton and at low doses, enhances the adhesion of *C. difficile* cells to the gastrointestinal epithelium by inducing the formation of microtubule-based protrusions in the host cell membranes [12,13,14]. Therefore, CDT might potentialize the toxicity of TcdA and TcdB and lead to more severe disease, which would be consistent with the correlation between the presence of binary toxin and the severe outcomes of CDI [9].

*C. difficile* infection (CDI) is a multi-step process that includes the disruption of the normal colonic microflora, the germination and outgrowth of spores (of endogenous or exogenous origin) and the subsequent colonization of the intestinal tract. During this process, vegetative cells multiply and produce both Toxin A and Toxin B, causing clinical manifestations, followed by the dissemination of spores. The spectrum of diseases caused by *C. difficile* was long thought to be related in part to the potential of strains to produce variable levels of toxins. Since that time, Akerlund’s group showed that toxin levels were correlated with the severity of *C. difficile* infection [15], confirming the existence of a relationship between the symptoms of *C. difficile*-associated diarrhea (CDAD) and the type and toxigenic potential of *C. difficile* strains [16]. In the early 2000s, this idea was reinforced by the emergence of the epidemic and hypervirulent *C. difficile* strain NAPI/027 [17,18], which was responsible for a massive increase in CDAD incidence and associated death. The major characteristic of this strain is that it produces larger amounts of toxins A and B than non-epidemic strains. Taken together, these observations strengthened the importance of the regulation of toxin synthesis for the pathogenicity of *C. difficile*. It is now well established that the levels of toxin production are influenced by growth conditions [19,20] and many environmental changes. One of the most important groups of environmental cues that influences toxin production is likely to be nutritional signals. For instance, limited concentrations of biotin or high levels of short-chain fatty acids in the medium increase toxin production [21], while rapidly metabolizable sugars, or certain amino acids, greatly reduce toxin yields [19,22,23,24,25,26]. Toxin production is also regulated by temperature, with an optimal temperature of 37 °C [27]. In addition, the impact of sub-inhibitory levels of antibiotics on toxin synthesis in *C. difficile* has also been widely investigated. Certain antibiotics appear to increase toxin production, but the specific response to an antibiotic appears to greatly vary from one strain to another [28,29,30,31,32,33,34].

To date, several regulation pathways that control toxin gene expression in response to several environmental and nutritional signals have been identified. In particular, the expression of the major virulence factors of *C. difficile* is under the control of a panel of nutrient-sensing regulators, suggesting that the regulation of toxin production must be an essential part of the adaptive strategies that are used by the bacteria in response to the environmental status encountered by *C. difficile* during infection. This review summarizes our current understanding of the signals and the mechanisms that regulate toxin synthesis in *C. difficile*.

## 2. The Pathogenicity Loci of *C. difficile* (PaLoc and CdtLoc)

**Figure 1:**
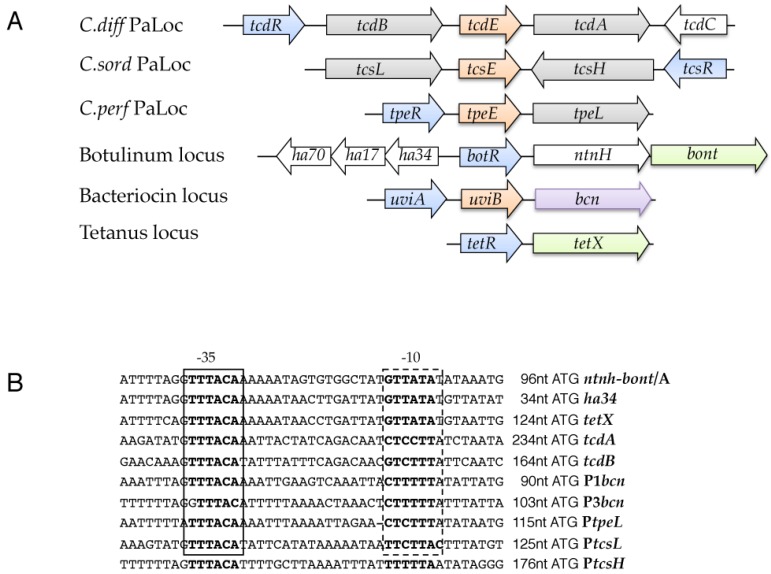
Toxin and bacteriocin loci regulated by the TcdR-family of σ factors in the pathogenic clostridia. (**A**). Genetic organization of clostridial toxin and bacteriocin encoding genes. Similarly colored genes encode functionally related proteins: large clostridial toxins in grey, neurotoxins in green, TcdR family alternative sigma factors in blue, holin-like proteins in orange and bacteriocin in purple; (**B**). Sequence alignment of the promoter regions of the toxin and bacteriocin genes. The −35 and −10 regions are based on determination of the transcriptional start sites [19,51,55,61,62]. The −35 box is highly conserved in promoters of all genes while the −10 box possesses greater variations.

One breakthrough in the understanding of the mechanism of toxin gene regulation originated from the molecular analysis of a 19.6 kb chromosomal region that is known as the pathogenicity locus (PaLoc) and is found only in toxigenic strains of *C. difficile* [35]. In addition to the genes that encode toxin A (*tcdA*) and toxin B (*tcdB*), the PaLoc contains three accessory genes: *tcdR* and *tcdC*, which encode proteins involved in the transcriptional regulation of the toxin genes [36,37], and *tcdE*, whose product is required for the efficient secretion of TcdA and TcdB [38] (Figure 1A). For the majority of strains, the PaLoc is found in the same genomic location and is replaced in non-toxigenic strains by a highly conserved 115/75 bp non-coding region [35,39]. However, it was recently shown that the PaLoc can be integrated in different genomic locations that are distant from the classical PaLoc integration site [40,41]. The presence of the PaLoc at different sites in the *C. difficile* genome suggested that the PaLoc was likely acquired by horizontal transfer, even if the PaLoc is not an independent mobile genetic element [35]. This hypothesis was supported by the G + C content of the PaLoc, which differs from that of the genome as a whole, and by the ability of the Paloc to be transferred to a non-toxigenic strain by a conjugation-like mechanism [42]. The recent identification of PaLoc-like regions in *C. sordellii* [43] and *C. perfringens* [44] that contain the LCT-encoding genes and *tcdR*- and *tcdE*-like genes supports the hypothesis that the LCT genes are located within PaLoc-related loci in multiple *Clostridium* species (Figure 1A).

A transcriptional analysis of the PaLoc indicated that the first four genes of the locus (*tcdR*, *tcdB*, *tcdE* and *tcdA*) are coordinately expressed when the cells approach stationary phase [20]. At this stage, the levels of *tcdA* mRNA are approximately two-fold higher than those of *tcdB* [19], which is consistent with larger amounts of TcdA being obtained after toxin purification [45]. Although *tcdA* and *tcdB* are transcribed primarily from their own promoters (see below), they can be found on a polycistronic transcript that is transcribed from a promoter identified >200 bp upstream of *tcdR* ORF [19,20,46].

CDT is encoded by two genes, *cdtA* and *cdtB*, which are transcriptionally linked and are located on the *C. difficile* chromosome in an operon at a locus called the CDT locus (CdtLoc) separate from the PaLoc. In most *C. difficile* strains that do not produce CDT, the CdtLoc is replaced by a 68-bp non-coding region. In contrast to the regulation of the PaLoc genes (see below), very little research has been carried out to understand how CdtLoc is regulated. However, the 6.2 kb CdtLoc includes a response regulator gene *cdtR*, located upstream the *cdt* operon. Carter *et al.* have shown that CdtR, a LytTR family response regulator, is able to activate CDT production [47]. This activation is likely direct since they have recently demonstrated the binding of purified CdtR to the upstream *cdtA* promoter region [48]. Further studies should focus on the nature of the unknown environmental or growth phase signals and on the histidine kinase sensor involved in the CdtR activation through the transfer of the phosphoryl group. Similar to the PaLoc, there is no evidence of transposon-, plasmid-, or bacteriophage-related genes in close proximity to the CdtLoc.

## 3. Toxin A and B Genes Are Specifically Transcribed by an Alternative Sigma Factor

The *tcdR* gene, which lies upstream of *tcdB* in the PaLoc, encodes a small basic protein of 22 kDa [35], which contains a C-terminal helix-turn-helix DNA-binding motif [36]. The first evidence for the role of TcdR in *C. difficile* toxin regulation was reported by Moncrief *et al.* [49]. Those authors successfully activated *tcdA* and *tcdB* reporter fusions by expressing *tcdR* *in trans* in *Escherichia coli* [49]. These results were confirmed in similar experiments that used *C. perfringens* [36] as a surrogate host and later in *C. difficile* [50]. Then, Mani and Dupuy provided genetic and biochemical evidence that TcdR is required for the initiation of toxin gene transcription as a σ factor of the RNA polymerase (RNAP) [36]. Indeed, TcdR is unable to bind alone to the toxin promoter regions but actually directs the RNAP core enzyme to specifically recognize promoters and transcribe toxin genes [36]. Interestingly, the promoter regions of *tcdA* and *tcdB* that show strong similarities to each other [19] do not resemble the canonical σ^70^ promoter of prokaryotes, which is consistent with the use of a specific σ factor, namely TcdR, for the transcription of toxin genes. In addition, TcdR also activates its own expression, which is consistent with the presence of two potential promoters for TcdR-dependent transcription in the region upstream of the *tcdR* gene [50].

TcdR is part of a new sub-group of the σ^70^ family that also includes other alternative σ factors of pathogenic clostridia that are required for the transcription of genes that encode the botulinum and tetanus neurotoxins (BotR and TetR, respectively [51]), the bacteriocin and cytotoxin of *C. perfringens* (UviA and TpeR, respectively [44,52]) and the lethal and hemorrhagic toxins of *C. sordellii* (TcsR [43]) (Figure 1A). In addition, all of the promoters of the genes encoding these toxins and bacteriocin share a high degree of sequence similarity with the *C. difficile* toxin gene promoters (Figure 1B) [52]. Many of the properties of these clostridial σ factors are similar to those of members of the extracytoplasmic function (ECF) σ factors (group IV of the σ^70^-family), but they differ enough in structure and function that they have been assigned to their own group (group V) [53]. Indeed, most of these TcdR-related σ factors are functionally interchangeable with each other but not with members of other sub-groups of the σ^70^ family [43,44,52]. Interestingly, these σ factors are encoded within genetic elements that were probably acquired by horizontal gene transfer; UviA, TetR, TcsR and TpeR are encoded on plasmids [44,54,55,56,57], TcdR is encoded within a PaLoc for which the presence of a putative holin and a phage-like endolysin fragment [58] suggest a bacteriophage origin [35] and BotR is encoded in bacteriophage genomes in some toxinotypes of *C. botulinum* [59]. In most cases, the genes that encode these σ factors are located immediately upstream of the promoters of toxin and bacteriocin genes and are transcribed in the same direction (Figure 1A). Moreover, in all examined cases, the σ gene is autoregulated [50,51,60] and induced by one or several environmental signals [19,27,44,50] (see below).

The similarities and the functional conservations within the group V σ factors reinforce the idea that the evolution of *Clostridium* species has been accompanied by the conserved use of a particular type of σ factor that is distinct from the ECF group to transcribe toxin and bacteriocin genes in the major pathogenic clostridia.

## 4. Toxin Gene Transcription Is Negatively Controlled by TcdC

The inverse timing of the expression of *tcdC* and the other PaLoc genes [20] has long suggested that TcdC could play a negative role in toxin gene expression. Moreover, the emergence of epidemic strains that produce high levels of both toxins and carry deletions or frameshift mutations in the *tcdC* gene reinforced the putative role of TcdC in the control of toxin production [17,63]. TcdC is not similar to any known regulatory protein and lacks a DNA-binding motif [64]. TcdC is an acidic, membrane-associated protein with a predicted molecular weight of 26 kDa [37,65]. This protein can form dimers, which is consistent with the presence of a coiled-coil motif in the middle of TcdC [37]. These biochemical characteristics suggested that TcdC could regulate toxin gene transcription as an anti-σ factor by modulating TcdR activity. Indeed, TcdC negatively regulates *C. difficile* toxin expression by interfering with the ability of the TcdR-containing RNAP to recognize toxin gene promoters. Both free TcdR and the preformed TcdR-containing holoenzyme were sensitive to TcdC activity. However, once a stable open complex was formed with the *tcdA* promoter, TcdC could not prevent the binding of the RNAP to the promoter [37], indicating that TcdC acts at the first stage of transcriptional initiation. TcdC sequesters TcdR in the manner of classical anti-σ factors but also interacts directly with the core RNAP, suggesting that TcdC may interfere with TcdR-dependent transcription via more than one mechanism [37]. Interestingly, TcdC was shown to inhibit transcription in a manner dependent on alternative σ factors, such as UviA and σ^W^, which is an ECF sigma factor, but not on primary σ^A^-type factors [37], suggesting that the mode of action of TcdC is more dependent on the nature of the σ factor than the core enzyme. Recently, van Leeuwen *et al.* [66] reported the ability of TcdC to bind to DNA folded into G-quadruplex structures containing repetitive guanine nucleotides, generally abundant in prokaryotic and eukaryotic gene promoters [67]. Thus, TcdC could also act by destabilizing open complex formation before transcription initiation. However, no quadruplex-forming motif with multiple G-stretches was found in the PaLoc raising the question of the relevance of such alternative function of TcdC in the control of toxin genes transcription.

Although these *in vitro* experiments clearly established that TcdC interferes with the TcdR-dependent transcription of toxin genes, recent *in vivo* studies generated contradictory results concerning the importance of TcdC for toxin expression. For instance, mutations within the *tcdC* genes are widespread among clinical isolates but cannot be used to predict the hyperproduction of toxins in these strains [68,69,70]. However, none of these conclusions were drawn from the analysis of isogenic *C. difficile* strains, which would be a prerequisite for clarifying the role of TcdC *in vivo*. Recently, Carter *et al.* generated an isogenic strain by introducing a plasmid-borne copy of *tcdC* into a *C. difficile* NAPI/027 strain (M7404) that lacks a functional *tcdC* gene. Those authors showed that the expression of TcdC within the native host results in the down-regulation of toxin production and an attenuated virulence phenotype in the hamster model of infection [71]. In contrast, another study showed that the chromosomal complementation of another NAPI/027 strain (R20291) with a functional *tcdC* gene did not change toxin titers [72] and that the disruption of the *tcdC* gene in the strain 630Δ*erm* had little if any effect on toxin production under the condition tested [72,73]. It was concluded in this study that TcdC might have a moderate role in regulating toxin expression and that TcdC is therefore not a major determinant of the hypervirulence of *C. difficile*. However, these studies did not exclude the possibility that TcdC could exert a more profound effect under specific conditions or in other strains of *C. difficile* than 630Δ*erm* and NAPI/027. Whatever the reason, further work is needed to conclusively define the role of TcdC in *C. difficile* and particularly on virulence capacity.

It has been clearly demonstrated that toxin synthesis is growth phase-dependent and regulated in response to several environmental signals. Therefore, several other regulatory factors must be involved in the control of toxin gene expression, either directly or indirectly through the modulation of TcdR synthesis or TcdR activity, at least by interacting with TcdC.

## 5. Toxin Expression Is Controlled by the Carbon Catabolite Repression (CCR) System

One of the most important groups of environmental cues that control toxin production is likely to be nutritional signals, such as carbon sources or certain amino acids. Different combinations of carbon sources that can be used by *C. difficile* to produce energy are available in culture media, which makes difficult the study of the detailed role of a particular substrate on toxin formation. It is likely that complex analysis to follow the temporal use and degradation pathways of the different carbon sources would help to further our knowledge of the regulatory network controlling toxin production [74]. However, despite this limit, addition of single substrates in culture media led to the emergence of an understanding of the associated regulatory mechanisms. The presence of glucose or other rapidly metabolizable carbon sources in a complex growth medium represses toxin production independently of the pH changes due to the glucose metabolism [19,22]. This glucose effect was observed in several *C. difficile* strains, suggesting that a general mechanism might be involved in the control of toxin production in response to glucose availability [19]. In addition, the repressive effect of glucose on toxin synthesis occurred at the transcriptional level, which was consistent with the repression of *tcdR* expression by glucose [50].

**Figure 2:**
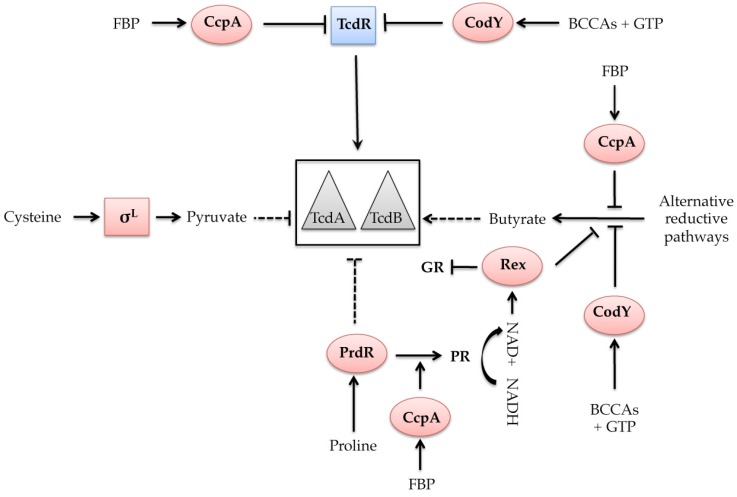
Effects of regulatory proteins and metabolites on *C. difficile* toxin synthesis. The metabolites that activate each regulatory protein is indicated: FBP, Fructose-1,6-bisphosphate; BCCAs, branch chain amino acids (Isoleucine, Valine, Leucine); NAD, Nicotinamide adenine dinucleotide. Alternative reductive pathways include glycine reductase (GR) pathway, butyrate production and succinate utilization pathway. Square boxes correspond to alternative σ factors while oval boxes are transcriptional regulators. Triangles are toxins. Arrowed lines indicate positive controls while lines ending with a bar across correspond to negative controls. Dashed arrows indicate mechanisms that are not fully understood.

Most carbon sources that repress toxin gene expression are transported inside the bacterium by the phosphoenolpyruvate-dependent carbohydrate: phosphotransferase system (PTS) [75]. This suggests that the regulation of toxin gene transcription by these rapidly metabolizable carbon sources involves carbon catabolite repression (CCR) [19]. The CCR allows bacteria to assimilate a preferred (e.g., rapidly metabolizable) carbon source, such as glucose, when exposed to more than one carbohydrate in the most profitable and economical way for the cell. In low G + C Gram-positive bacteria*,* the main mechanism of the CCR is mediated by the pleiotropic regulator CcpA, which is a member of the LacI/GalR repressor family. CcpA usually acts as a repressor of the utilization of alternative carbon sources and as a positive regulator of pathways associated with glycolysis. Positive and negative transcriptional control by CcpA involves the binding of this regulator to a *cis*-acting catabolite responsive element (*cre*) that is located upstream or in the 5′ part of the target genes. Moreover, the DNA binding activity of CcpA to the *cre* sites is enhanced by its interaction with a component of the PTS, the HPr protein. To activate CcpA, HPr must be phosphorylated at the regulatory residue Ser-46 by the HPr kinase/phosphorylase (HPr-K/P) according to the intracellular concentration of fructose-1-6-phosphate (FBP) [76,77,78]. Using *C. difficile* mutant strains defective in genes of the PTS or in *ccpA*, it has been shown that both the uptake of glucose and the global regulator CcpA are required for glucose-dependent repression of toxin genes [24]. In fact, only 50% of the glucose-regulated genes are controlled by CcpA, whereas it is >80% in *B. subtilis* [79]. In *C. difficile,* the CcpA regulon includes genes involved in sugar uptake, fermentation and amino acid metabolism, confirming the role of CcpA as a link between carbon and nitrogen pathways and virulence gene expression in *C. difficile*. CcpA mediates glucose-dependent repression of toxin production by interacting directly with the promoter region or the 5′ ends of several PaLoc genes, with the strongest affinity for the promoter region of *tcdR* [79] (Figure 2). This observation is consistent with the presence of two *cre* sites upstream of the transcriptional start of *tcdR*, whose sequence is significantly different from the consensus *cre* site defined in *Bacillus subtilis* [79]. In addition, in contrast to FBP alone, neither HPr nor HPr-Ser-64-P stimulated CcpA binding to its targets, which differs from the classical mode of action of CCR in *B. subtilis* [24]. Interestingly, toxin production in the *C. difficile* *ccpA* mutant strain is constitutive with respect to glucose but lower than that observed in the wild-type strain without glucose. This finding indicates that other regulator(s) controlled by CcpA must contribute(s) to the regulation of toxin gene expression independently of glucose. In *C. perfringens*, CcpA is known to regulate the expression of enterotoxin (CPE) in a growth phase-dependent manner, which is not associated with the presence of glucose [80]. Thus, it appears that the regulation of *C. difficile* toxin synthesis in response to glucose availability is the result of a complex regulatory network in which CcpA plays a central role. Indeed, CcpA controls directly or indirectly a large number of regulators [79] and participates with other global regulators, such as CodY and Rex, in coordinating metabolism and virulence through the regulation of fermentation processes that produce butyrate, which is known to stimulate toxin production (Figure 2 and see below). It was recently shown that glucose also repressed the synthesis of LCT by *C. sordellii* and *C. perfringens* [43,44]. Both *C. sordellii* and *C. perfringens* encode CcpA homologs, but their role in the glucose-dependent regulation of toxin production has not yet been demonstrated.

## 6. Regulation of Toxin Synthesis in Response to Proline and Cysteine Availability

Proline and glycine have a powerful inhibitory effect on toxin production*.* The impact of both of these amino acids on the *C. difficile* toxin yield [22] suggested a role for Stickland metabolism in the control of toxin production. Like many *Clostridium* spp., *C. difficile* uses the Stickland reaction, which involves the coupled oxidation and reduction of pairs of amino acids to generate ATP and NAD^+^. The oxidative path way generates ATP and NADH, while the reductive pathway regenerates NAD^+^ from NADH. Proline reductase (PR) and glycine reductase (GR) are specifically induced in the presence of proline and glycine, respectively, and carry out the respective reduction of these amino acids [81]. Moreover, the addition of proline to the growth medium decreases the expression of the GR-encoding genes, suggesting a preferential utilization of proline for NAD^+^ regeneration [26]. PrdR, which is a regulator that responds to proline, mediates both the proline-dependent activation of PR and the proline-dependent repression of toxin genes and the GR operon (Figure 2) [26]. However, nothing is known regarding the mode of action of PrdR (direct or indirect) in the control of the PR, GR and toxin gene expression. The global effect of proline and PrdR on *C. difficile* gene expression was recently studied via transcriptomic analyses and most of the proline-dependent effects on gene expression appeared to be mediated by PrdR. The genes whose expression was strongly affected are involved in alternative pathways of NAD^+^ regeneration, such as GR, butyrate production and the succinate utilization pathway, suggesting a hierarchical control of NAD^+^ regeneration [82]. Thus, when proline is limiting in the medium or if PrdR or PR is inactive, the alternative reductive pathways are induced. In fact, both PrdR and a functional PR are indirectly required for the proline-dependent regulation of the alternative reductive pathways in response to the intracellular concentration of NADH and NAD^+^. This process involves the global redox-sensing regulator Rex [82]. In several Gram-positive bacteria, Rex acts as a repressor of genes that are important for growth using fermentation [83,84,85,86,87,88]. Rex directly senses changes in redox status and is only active as a DNA-binding protein when the intracellular NADH/NAD^+^ ratio is low [84]. A Rex homolog is present in *C. difficile*, and putative Rex boxes are identified upstream of PrdR-regulated genes, including those involved in the fermentation pathways that produce butyryl-CoA from acetyl-CoA or succinate [89]. In a *rex* null mutant, the addition of proline no longer represses these pathways. Moreover, the purified Rex protein can bind to the respective genes, and the binding activity of Rex is stimulated by NAD^+^ but inhibited by NADH (Figure 2) [82]. Although Rex, like PrdR, controls the proline-responsive expression of these alternative reductive pathways, Rex also mediates the proline-dependent repression of toxin gene expression, probably through the regulation of butyrate production. Consistent with this model, PrdR is active and stimulates PR expression when proline is in excess. As a result, the NADH/NAD^+^ ratio is low and Rex is active as a repressor of the alternative NAD^+^ regeneration pathways. In contrast, if proline becomes limiting, the NADH/NAD^+^ ratio increases and NADH prevents Rex-dependent repression of the alternative pathways. The regeneration of NAD^+^ using these alternative reductive pathways leads to an accumulation of butyrate, a compound that stimulates toxin synthesis (Figure 2).

Cysteine is the most potent amino acid that down-regulates toxin production in both the reference strain 630 and clinical isolates [23,25]. This sulfur-containing amino acid plays a central role in bacterial physiology. Cysteine is a precursor of methionine and several co-enzymes, a sulfur donor for the biogenesis of iron-sulfur clusters and is found in the catalytic sites of several enzymes. Links between cysteine metabolism and bacterial virulence have been described in several pathogenic bacteria, including *C. perfringens* and *Bordetella pertussis* [90,91]. To understand the molecular mechanism involved in the cysteine-dependent repression of toxin production, a global transcriptomic analysis of the genes controlled by cysteine was recently performed in *C. difficile* [92]. Genes that are regulated in response to cysteine availability are involved in amino acid and energy metabolism, as well as in fermentation pathways. It was initially demonstrated that cysteine does not regulate toxin production by acting as a reducing agent [22]. Because cysteine metabolism during cell growth has an impact on the pools of several metabolites, including carbon sources and amino acids, some global regulators and σ factors, such as CcpA, CodY and σ^H^, that are known to regulate toxin gene expression were tested [24,93,94]. Among those factors, only σ^L^, which belongs to the σ^54^ family and plays an important role in the metabolism and virulence of Gram-positive bacteria [95,96], was shown to mediate cysteine-dependent repression of toxin production (Figure 2) [92]. Because no σ^L^-type promoter was found upstream of toxin genes, it was hypothesized that σ^L^ regulates the PaLoc genes indirectly, probably in response to by-products of cysteine degradation. This hypothesis was consistent with the role of σ^L^ in the control of cysteine degradation in *C. difficile*. Moreover, the addition of the first by-products of cysteine (pyruvate and sulfide) during cell growth decreased the transcription of toxin genes and *tcdR* in both the wild-type and *sigL* mutant strains. In contrast, the addition of other pyruvate by-products, such as formate and acetate, had no effect on PaLoc gene transcription [92]. Thus, pyruvate and sulfide, rather than cysteine, might be the main signals that modulate toxin production (Figure 2). Further studies will be necessary to characterize the mechanism involved in toxin production in response to cysteine by-products.

## 7. Regulation of Toxin Synthesis by Nutritional Limitation Is Mediated by CodY

A strong link between nutrient limitation and toxin production came from the discovery that the expression of all of the PaLoc genes is repressed by the global transcriptional regulator CodY, which is involved in the adaptive response to nutrient sufficiency in the environment [93]. CodY is highly conserved in low G + C content Gram-positive bacteria. In *B. subtilis*, during rapid growth, CodY represses more than 100 genes whose products are not needed when nutrients are in excess and releases this repression when nutrients become limited at the onset of stationary phase [97]. CodY targets are associated with a range of adaptive behaviors, such as sugar and amino acid transport [97], mobility [98] and sporulation [99]. In some pathogenic bacteria, CodY controls not only genes involved in major physiological processes but also virulence factors [100]. CodY is a dimeric protein that binds to DNA through a winged helix-turn-helix motif that is located in the C-terminal part of the protein. The binding affinity of CodY for its target genes is increased in the presence of branched-chain amino acids (isoleucine, leucine, and valine (BCAAs)) and GTP, either individually or in combination [99,101]. The presence of these cofactors is a signal of nutrient availability. Thus, when nutrients become limiting, the intracellular levels of BCAAs and GTP are reduced, and CodY is no longer able to repress genes involved in bacterial adaptation to starvation.

**Figure 3:**
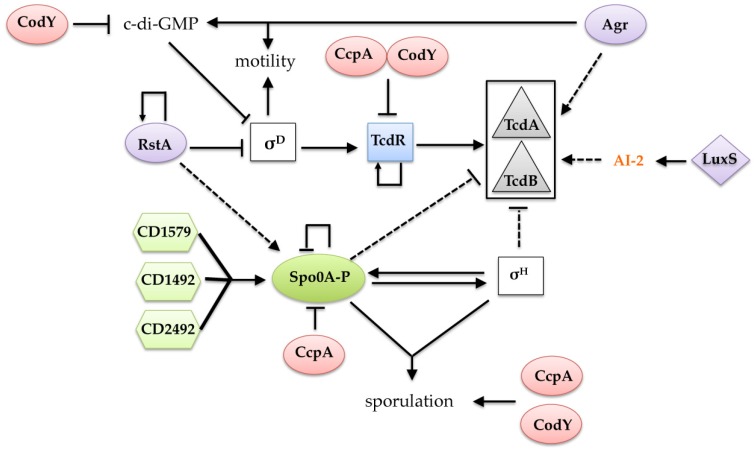
The regulatory network of the transition phase and the initiation of sporulation controlling toxin production in *C. difficile*. The regulatory factors likely involved in quorum sensing are indicated in purple and those involved in the response to the nutritional status of the cell are indicated in red. Spo0A and its associated kinases (CD1579, CD1492, CD2492) are indicated in green. Square boxes correspond to alternative σ factors while oval boxes are transcriptional regulators and diamond box is enzyme. Arrowed lines indicate positive controls while lines ending with a bar across correspond to negative controls. Dashed arrows indicate mechanisms that are not fully understood. AI-2 and c-di-GMP mean auto-inducer 2 and cyclic di-guanosyl-5′monophosphate.

In *C. difficile*, more than 140 genes are under the control of CodY. Most of these genes are involved in the biosynthesis of amino acids, nutrient transport fermentation pathways, sporulation and toxin production [102]. Phenotype analysis of a *C. difficile* *codY* mutant showed that all of the PaLoc genes are strongly derepressed during exponential phase [93], which is consistent with the higher level of toxin produced in the *codY* mutant in comparison to the wild-type strain [82]. Indeed, CodY regulates toxin gene expression by binding to the *tcdR* promoter region at three different locations with varying affinities; two of these locations overlap with σ factor-binding regions [93]. Like in *B.* subtilis, GTP and BCAA are co-factors of CodY in *C. difficile* (Figure 2) and exert independent and additive effects on the *in vitro* activity of CodY [93]. This observation supported previous evidence that the presence of nine amino acids in the medium, including BCAAs, significantly reduced *C. difficile* toxin synthesis [22]. Interestingly, CodY can interact with other regulatory networks in the control of both metabolic and toxin genes. This type of interaction probably occurs through cyclic-di-GMP (c-di-GMP) signaling, which is an important signal transduction system in the lifestyle of this pathogen (Figure 3). C-di GMP is synthesized from 2 GTP molecules by diguanylate cyclases (DGCs) and degraded into pGpG or 2 GMP by phosphodiesterases (PDEs). The pool of c-di-GMP is controlled by a plethora of DGC and PDE enzymes, and genes encoding several of these enzymes are controlled by CodY [102,103]. Thus, changes in the intracellular concentration of GTP correlated with c-di-GMP synthesis can modify the CodY activity. In addition, based on nutrient availability, CcpA can stimulate the production of BCAAs, which are the second effector of CodY, illustrating that the regulatory pathways of CodY and CcpA are intertwined. Finally, CodY, which directly represses PaLoc genes, can also contribute to the repression of toxin synthesis by regulating the synthesis of butyrate (Figure 2). In fact, all enzyme-encoding genes that are required for butyrate synthesis from acetyl-CoA and succinate are controlled by CodY in response to the levels of BCAAs and GTP, as Rex responds to the NADH/NAD^+^ ratio (Figure 2).

## 8. The Regulatory Network That Controls Transition Phase, Sporulation and Toxin Production in *C. difficile*

*In vitro,* toxin synthesis increases when the cells enter stationary phase [19], as was also observed *in vivo* in the mouse model of CDI [104]. It was recently shown that toxin production is controlled by regulators of the complex-regulatory network that is set up at the onset of stationary phase and involved in the control of the initiation of sporulation [94,105,106,107]. The regulatory mechanisms of the transition from exponential growth phase to stationary phase have been studied extensively in *B. subtilis* [108], but differences exist in *C. difficile* [94,109,110]. The initiation of sporulation in *B. subtilis* is controlled by a regulatory cascade (phosphorelay) that consists of five sensor kinases, two intermediary phosphorylated proteins (Spo0B and Spo0F) and several phosphatases [111,112]. These proteins modulate the level of phosphorylation of the response regulator Spo0A in response to nutrient availability, cell density and other signals. The phosphorylated form of Spo0A then activates the transcription of several key sporulation genes. In addition, the alternative σ factor SigH, which is involved in the transcription of major genes of the transition phase, is also required for the expression of *spo0A* [113]. Both Spo0A and SigH are present in *C. difficile,* but orthologs of most of the phosphorelay proteins, including Spo0B, Spo0F and the phosphatases, are missing. In the clostridia, the sporulation initiation pathway is a two-component system with associated kinases that directly phosphorylate Spo0A [114,115]. Three orphan histidine kinases are present in the *C. difficile* genome (CD1492, CD2492, and CD1579) (Figure 3). CD1579 phosphorylates Spo0A *in vitro,* while the inactivation of *CD2492* reduces the sporulation efficiency of the strain 630Δ*erm* [110]. As observed in *B. subtilis* [113,116], SigH and Spo0A of *C. difficile* regulate not only sporulation genes but also numerous genes that are involved in several functions, such as metabolism, including the butyrate biosynthesis pathways, cell wall metabolism, motility or encoding surface-associated proteins [94,105]. Moreover, SigH and Spo0A control toxin gene expression at the onset of stationary phase (Figure 3). In a *sigH* mutant, the *tcdA*, *tcdB* and *tcdR* genes were overexpressed, implying that SigH represses toxin expression through a mechanism that is likely indirect, as no SigH promoter has been found upstream of any of the PaLoc genes [94]. Thus, SigH may act either by controlling the transcription of a gene that encodes a repressor of toxin gene transcription or by competing with TcdR for interaction with the core enzyme of the RNAP. The effect of *spo0A* inactivation on toxin production appears to be strain-specific. Indeed, Spo0A represses toxin gene expression in some strains of ribotype 027 [106,107] but has no or marginal effects in strains of ribotype 078 [106] and conflicting results have been reported for the role of Spo0A on toxin production in 630Δ*erm* [105,106,107,110,117]. In addition, none of the promoters of the PaLoc genes contains a Spo0A binding sequence, suggesting that the effect of Spo0A on toxin gene expression is indirect and needs to be characterized. Further work will be required to decipher the molecular mechanisms that lead to the control of toxin production by Spo0A and SigH and to identify the involved regulator(s) among those genes that are under the control of Spo0A and/or SigH in transcriptomes [94,105].

Recently, a new regulator (RstA) that positively controls sporulation initiation and negatively affects mobility and toxin production was identified [118]. RstA regulates toxin production and mobility by repressing the transcription of SigD, the flagella-specific σ factor, which in turn directly controls *tcdR* transcription (see below, Figure 3). RstA controls sporulation initiation through an unidentified pathway, which is SigD-independent. This regulator belongs to the RNPP family of transcriptional regulators involved in quorum sensing that includes PlcR, NprR and PrgX [119]. Moreover, RstA is conserved in pathogenic and non-pathogenic clostridia and closely related organisms, suggesting that RstA is a key regulator protein that controls both sporulation and virulence functions in these bacteria.

Reports that describe correlations between spore formation and toxin gene regulation in *C. difficile* are contradictory [15,22,23,120]. In *C. perfringens,* sporulation and enterotoxin synthesis (Cpe) are controlled by the mother cell-specific sigma factors SigE and SigK [121]. Indeed, the *cpe* gene is specifically transcribed during sporulation from SigK- and SigE-dependent promoters, and its expression was completely abolished in a *sigE* mutant [121,122]. In *C. difficile*, the *sigH* mutant is unable to sporulate but still produces toxins. This finding indicated that sporulation is not required for toxin synthesis and is instead a stationary-phase event. Consistent with this observation, no transcriptional control of toxin genes by the four sporulation-specific sigma factors (SigF, SigE, SigG and SigK) was observed in transcriptome analyses [123,124], and no toxins were detected in the proteome of the spore [125] or the outer layers of the spore [126]. However, recent work using more targeted approaches suggested that *tcdA*, *tcdB* and *tcdR* are expressed during sporulation [127]. Thus, toxins might be produced not only at the onset of stationary phase after bacterial multiplication during the colonization process [104] but also later during spore formation, leading to the production of spores that contain toxins. Further work will be necessary to determine the extent to which toxinogenesis and sporogenesis overlap and the impact of the possible relationship between these two processes that play a central role in the *C. difficile* infectious cycle.

## 9. Other Regulators That Control Toxin Production

### 9.1. Toxin Gene Expression Is Controlled by Quorum Sensing

Quorum sensing (QS) is a cell density-dependent mechanism of gene expression control that involves signaling molecules termed auto-inducers (AIs). Many virulence factors are produced by bacterial pathogens at high cell density through quorum signaling systems. In Gram-positive bacteria, these cell-cell communication systems involve peptides called autoinducing peptides (AIP), which act as signaling molecules. In *Staphylococcus aureus*, the *agr* quorum-sensing locus *agrACDB* is involved in controlling the expression of many secreted virulence factors during the transition phase [128]. The biosynthesis of AIP requires AgrD, which is the peptide precursor of AIP, and AgrB, which is a transmembrane endopeptidase involved in the processing and exportation of AIP. The accumulation of extracellular concentrations of AIP activates a two-component system (TCS) by binding to the sensor histidine kinase AgrC, which in turn modulates the activity of the response regulator AgrA. All analyzed *C. difficile* genomes contain an incomplete *agr*- locus that contains *agrDB* and is named the *agr1* locus [129]. However, in addition to *agr1*, a complete *agrACBD* operon (*agr2* locus) is present in the genome of the *C. difficile* ribotype 027 isolate R20291 and in the genomes of some clinical strains [129,130]. In strain R20291, the inactivation of *agrA* results in decreased toxin production (Figure 3) and this mutant exhibits a colonization defect in mice [131,132]. Interestingly, AgrA also controls flagellar synthesis and the expression of genes involved in c-di-GMP metabolism [132] that are known to influence toxin gene expression, as indicated below. Thus, the Agr2 system appears to play a role in *C. difficile* pathogenesis, at least in some lineages. In the 630 and R20291 backgrounds, a thiolactone peptide that is likely synthesized by the *agr1* locus is also involved in the induction of toxin production [131]. The AgrD1 and AgrD2 prepeptides, which are encoded by the *agr1* and *agr2* loci, respectively, are 34% identical, but a cysteine residue that is probably involved in thioester bond formation is only present in AgrD1. The TCS that senses AgrD1 remains to be identified. Interestingly, an *agrBD* locus that is not linked to a TCS is also present in *C. perfringens.* This Agr system positively controls the expression of various toxin genes in type A, B, C and D strains of *C. perfringens* [133,134,135,136,137]. In *C. acetobutylicum,* a complete *agrBDCA* cluster is involved in the cell density-dependent regulation of granulose production and spore formation [138]. Two *agrBD* loci are also present in *C. botulinum*. Both loci control sporulation and toxin production, with a more drastic effect on neurotoxin synthesis for AgrD2 [139]. In *C. difficile,* further work will be needed to decipher the regulatory mechanisms associated with the two Agr systems, to study the relationship between Agr1 and Agr2 and to investigate the possible involvement of Agr1 in the control of sporulation.

In addition to the intra-species communication that is mediated by AIP in Gram-positive bacteria, interspecies communication is also effective and involves a second class of QS molecules (*i.e.*, autoinducer-2 (AI-2)) that has been identified in several bacterial species [140]. The LuxS enzyme, which forms part of the methyl cycle pathway, is involved in AI-2 synthesis from S-ribosylhomocysteine [140,141]. Many pathogens, including *C. difficile,* have QS systems that detect AI-2 and are involved in virulence expression. AI-2 up-regulates the expression of PaLoc genes (*tcdA*, *tcdB* and *tcdE*) when added to the culture medium early during growth (Figure 3) [142]. However, this effect is not observed in samples harvested after 24 h and 48 h of growth [143]. Interestingly, it has been reported that a major catechin compound (Epigallocatechin gallate) isolated from green tea downregulates LuxS production, leading to a decrease in the amount of AI-2 and lower expression of the *tcdA* and *tcdR* genes [144]. In *C. perfringens*, LuxS/AI-2 also contributes to the regulation of α, κ and θ toxin expression [145]. Further work will be necessary to decipher the molecular mechanisms involved in the transient effect of AI-2 on toxin gene expression at the onset of stationary phase.

### 9.2. Control of Toxin Expression by c-di-GMP

The signaling molecule cyclic di-guanosyl-5′monophosphate (c-di-GMP) is a second messenger in bacterial systems and a key feature in the control of critical lifestyle choices, such as the transition between planktonic and biofilm growth. Thus, elevated levels of c-di-GMP typically promote sessile lifestyles, such as biofilm formation, while low levels of c-di-GMP are associated with motility [146]. In contrast to most Gram-positive bacteria, including some close relatives, *C. difficile* encodes a large number of c-di-GMP turnover enzymes (18 predicted diguanylate cyclases and 19 predicted phosphodiesterases), and enzymatic activity was confirmed for many of those enzymes [103,147]. In addition, 16 riboswitches that respond to c-di-GMP have been identified in *C. difficile* [148], underlining the importance of c-di-GMP signaling in this human pathogen. In recent years, c-di-GMP was found to regulate classical functions in *C. difficile*, including swimming and twitching motility, adhesion, aggregation and biofilm formation [148,149,150,151,152,153]. Surprisingly, c-di-GMP was also shown to affect toxin gene expression [154]. Indeed, artificial elevation of intracellular levels of c-di-GMP in *C. difficile* strongly represses the transcription of toxin-encoding genes and *tcdR* [154]. Interestingly, the presence of high levels of c-di-GMP transcription also inhibited the expression of the gene encoding the flagellar alternative σ factor SigD [154]. Moreover, in a *sigD* mutant, the expression of the *tcdA, tcdB* and *tcdR* genes was reduced, and it was demonstrated that SigD directs the RNAP core enzyme to recognize the *tcdR* promoter but not the *tcdA* and *tcdB* promoters [155]. Thus, the c-di-GMP-dependent regulation of toxin expression is indirect and mediated by SigD, which is involved in *tcdR* transcription and consequently in toxin synthesis (Figure 3).

### 9.3. Regulation of Toxin Expression by Flagellar Proteins

In addition to SigD, other flagellar components, such as the flagellin FliC and the capping protein FliD, influence toxin production in *C. difficile*. In strain 630Δ*erm*, the inactivation of *fliC*, which is a late-stage flagellar regulon gene, led to increased expression of all Paloc genes except *tcdC* [156]. This result was reinforced by another study that showed that the levels of *tcdA* transcripts are increased in both *fliC* and *fliD* mutants in the same background [157]. Consistent with these data, the activity of toxins in cell culture supernatants of *fliC* and *fliD* mutants was increased in comparison to the parental strain. Moreover, these mutants were reported to be more virulent in hamsters in some but not all studies [158]. In addition, toxin expression is not significantly altered in a strain lacking the glycosyltransferase CD0240, which is an enzyme involved in FliC glycosylation [156]. The inactivation of *CD0240* resulted in cells that are non-motile, although unglycosylated FliC is still produced and exposed at the cell surface [159]. Therefore, it appears that the loss of the flagellin FliC rather than a non-functional flagellar filament affects toxin expression. It has been noted that the epidemic strain R20291 produces only a single flagellum that is required for adherence to CaCo-2 cells, whereas strain 630Δ*erm* is peritrichously flagellated, with flagella that contribute to overall fitness. Interestingly, in contrast to strain 630Δ*erm*, no change in cytotoxicity was observed between the R20291 wild-type strain and the isogenic flagella mutants [157]. In addition, *in vivo* transcriptomic analysis performed at 14 h post-infection revealed that toxin transcription was not affected in a *fliC* mutant in comparison to the R20291 wild-type strain. Thus, it appears that the influence of flagella on toxin production differs depending on the *C. difficile* strain and is related to the structural components of the flagellum. More work is needed to better understand the exact involvement of the flagellar regulon in toxin expression.

### 9.4. Impact of the Presence of Prophages on Toxin Gene Expression

Bacteriophages are the most abundant and diverse entities in the biosphere. Bacteriophages can influence the abundance, diversity and evolution of bacterial communities [160]. Some lysogenic phages can also modify the phenotypes of their hosts by encoding key virulence factors, such as the diphtheria and botulinum toxins [161]. *C. difficile* has been shown to contain a number of different bacteriophages [162], and the lysogenization of some of these bacteriophages has been shown to affect toxin synthesis [163]. Indeed, it was shown that in the ϕCD119 or ϕCD27 lysogens derived from *C. difficile* isolates, toxin production is repressed in comparison to the respective parental strains [164,165]. In the case of ϕCD119, the expression of all PaLoc genes is downregulated in the lysogenic strain in comparison to the parental strain, and this expression modulation involves the phage-encoded protein RepR [164]. Furthermore, reporter gene fusion experiments and DNA binding studies showed that RepR decreases toxin production indirectly by controlling the transcription of *tcdR*.

In contrast, the lysogenization of a ribotype 027 strain by ϕCD38-2 results in the stimulation of toxin production [166]. In fact, in comparison to its parental strain, the level of toxin in a ϕCD38-2 lysogenic strain was significantly increased in the culture supernatant but not in the cytosol. Interestingly, the transcription of all PaLoc genes was higher in the ϕCD38-2 lysogen strain, but *tcdE* appears to be expressed more strongly than the other genes of the PaLoc. The *tcdE* gene encodes a protein that is similar to a class of bacteriophage proteins named holins that is required for the efficient release of TcdA and TcdB from bacteria [38]. Together, these results suggested that the lysogenic strain carrying ϕCD38-2 synthesizes and secretes more toxins than the parental strain due to the increased expression of PaLoc genes, especially *tcdE*. Further experiments will be necessary to understand how the ϕCD38-2 can influence toxin gene expression. An increase in toxin production was also described in other lysogens of *C. difficile* strains carrying the prophages ΦC2, ΦC6 and ΦC8. In these cases, the effect likely relied on the modulation of toxin production or release rather than on the transcription of *tcdA* and *tcdB* [167]. Thus, these reports raised the possibility that cross-talk between prophages and bacterial genes exist in *C. difficile,* as illustrated by their impact on the PaLoc genes.

### 9.5. Links between the Stress Response and Toxin Gene Expression

The SOS regulatory network plays a central role in the bacterial response to DNA damage, which is controlled by the global repressor LexA and an inducer, the recombinase RecA [168]. During normal bacterial growth, LexA binds to the promoter region of the SOS genes and prevents their transcription. Upon DNA damage, RecA polymerizes on single-stranded DNA that has formed at sites of DNA damage and becomes activated. The activated form of RecA facilitates the inactivation of LexA via self-cleavage, resulting in the expression of SOS genes [169]. In *C. difficile* strain R20291, the *lexA* mutant exhibits alterations in cell division, motility, and sporulation and has a greater sensitivity to metronidazole [170]. Moreover, LexA negatively controls biofilm formation. In the presence of sub-inhibitory concentrations of levofloxacin, the production of TcdA but not TcdB increased in a *lexA* mutant in comparison to the wild-type strain [170]. Levofloxacin is known to induce the SOS response [171] and to repress toxin production in *C. difficile* [31,172]. Thus, these data suggested that the inhibitory effect of levofloxacin on TcdA synthesis was countered by the constitutively induced SOS response that is observed in the absence of LexA. Changes in the regulation of TcdA but not TcdB in the *lexA* mutant are somehow consistent with the ability of LexA to bind only to the *tcdA* promoter region, which contains a LexA binding motif [173]. Thus, LexA not only regulates DNA damage but also controls other biological functions, including the regulation of toxin A production.

The transcription-repair coupling factor (Mfd in bacteria) is a highly conserved bacterial protein that links the processes of nucleotide excision and transcription elongation [174]. In response to DNA damage, Mfd removes RNA polymerase that is stalled at DNA lesions, increasing the rate at which the lesions are repaired [175]. The removal of stalled RNAP is not confined to DNA lesions but also occurs for RNAP that is stalled for other reasons, such as nucleotide starvation or blockage by other DNA proteins, such as transcriptional repressors [176]. The inactivation of *mfd* in *C. difficile* resulted in an unusual colony morphology and increased expression of TcdA and TcdB, which occurs at the transcriptional level [177]. In *B. subtilis*, Mfd inactivation partially relieved the CodY- or CcpA-mediated transcriptional repression of genes with binding sites downstream of the promoters [178,179,180]. Because both CcpA and CodY binding sites have been identified downstream of the *tcdR* promoter [79,93], it is possible that Mfd can affect the transcriptional regulation of toxin genes by relieving RNA polymerase that is stalled at roadblocks created by CodY and/or CcpA, which are two major repressors of toxin gene expression in *C. difficile* (Figure 3).

## 10. Conclusions

In the murine model, toxin synthesis occurs late during infection [104]. The complex regulation of the *C. difficile* PaLoc genes that involves multiple environmental and physiological factors, suggests that the bacterium’s strategy to persist and cause damage to the host is closely related to its nutritional status. In response to transition phase, nutrient limitation and stresses, as well as the detection of cell density through quorum sensing in the gastrointestinal tract during dysbiosis, the regulatory network involved must contribute to trigger toxin production when needed. Particularly, the crucial role of the control of toxin production in the response to nutrient limitation indicates that an intimate connection exists between virulence and metabolism. Thus, the use by *C. difficile* of several global metabolic regulators, such as CcpA, CodY, SigL, PrdR and Rex, to control toxin genes expression implies from the bacterium‘s point of view that virulence is to a great extent a mechanism for improving nutrient availability. Accordingly, such coordination is illustrated by the impact of butyrate on the regulation of toxin synthesis. Indeed, in addition to toxin synthesis, the major global regulators CodY and CcpA control the expression of the alternative reductive pathways that regenerate NAD^+^ [24,93]. That is, in response to FBP, CcpA activates the expression of the proline reductase gene cluster and represses both the alternative NAD^+^-regenerating pathways that lead to butyryl-CoA and the genes (*ptb* and *buk*) encoding enzymes that are used to convert butyryl CoA to butyrate [24]. These pathways are also controlled by CodY in response to BCAAs and GTP [102]. Thus, when cells have an excess of NADH, butyrate is produced suggesting that the stimulation of toxin synthesis by butyrate is a response to the redox status of the cell (NADH/NAD^+^ ratio) and nutrient limitation (*i.e.*, proline, BCAAs, and carbohydrates). The molecular mechanism by which butyrate activates toxin synthesis remains to be discovered. Interestingly, many inhabitants of the lower gastrointestinal tract produce butyrate, while pyruvate, which down-regulates toxin production is apparently more abundant in the upper part of the gut. This raises an interesting question about the role of both butyrate and pyruvate and the mechanisms involved in the control of toxin synthesis along the intestinal tract in the context of gut dysbiosis. Furthermore, the butyrate precursor butyril CoA, is also the precursor of butanol, an inhibitor of toxin synthesis [25]. Thus, it would be interesting to know how the bacterium decides to convert butyryl CoA into butanol or butyrate that would potentially help for the development of novel therapeutics to reduce virulence. Finally, as virulence factors other than toxins become identified, it will be also interesting to determine how they are controlled and to which environmental signals they respond in order to better evaluate the extent to which *C. difficile* pathogenesis is influenced by intracellular metabolite pools.
